# NGAL as an Early Predictive Marker of Diabetic Nephropathy in Children and Young Adults with Type 1 Diabetes Mellitus

**DOI:** 10.1155/2017/7526919

**Published:** 2017-05-15

**Authors:** Nektaria Papadopoulou-Marketou, Alexandra Margeli, Ioannis Papassotiriou, George P. Chrousos, Christina Kanaka-Gantenbein, Jeanette Wahlberg

**Affiliations:** ^1^Department of Endocrinology, Department of Medical and Health Sciences, Linkoping University, Linkoping, Sweden; ^2^Diabetes Centre, Department of Endocrinology, Diabetes and Metabolism, First Department of Pediatrics, National and Kapodistrian University of Athens, Aghia Sophia Children's Hospital, Thivon, 115 27 Athens, Greece; ^3^Department of Clinical Biochemistry, Aghia Sophia Children's Hospital, Thivon, 115 27 Athens, Greece

## Abstract

**Aims:**

Type 1 diabetes (T1D) is often associated with early microvascular complications. Previous studies demonstrated that increased systolic (SAP) and diastolic arterial blood pressures (DAP) are linked to microvascular morbidity in T1D. The aim of the study was to investigate the predictive role of neutrophil gelatinase-associated lipocalin (NGAL) in unravelling early cardio-renal dysfunction in T1D.

**Methods:**

Two T1D patient groups participating in two-centre prospective cohorts were studied. Group A consisted of 57 participants aged 13.9 years (SD: 3.1) and group B consisted of 59 patients aged 28.0 years (SD: 4.4). Forty-nine healthy children [age: 10.5 years (SD: 6.6)] and 18 healthy adults [age 27.7 years (SD: 4.2)] served as controls. Serum concentrations of NGAL (ELISA) were determined, and SAP and DAP were examined (SAP and DAP also expressed as *z*-scores in the younger group).

**Results:**

NGAL correlated positively with SAP in both patient groups (*P* = 0.020 and *P* = 0.031, resp.) and SAP *z*-score (*P* = 0.009) (group A) and negatively with eGFR in both groups (*P* < 0.001 and *P* < 0.001, resp.).

**Conclusions:**

NGAL may be proposed as a biomarker of early renal dysfunction even in nonalbuminuric T1D patients, since it was strongly associated with renal function decline and increasing systolic arterial pressure even at prehypertensive range in people with T1D, in a broad age range.

## 1. Introduction

Type 1 diabetes mellitus (T1D) is a prevalent autoimmune disease in childhood and young adulthood. Diabetes nephropathy (DN) is a chronic devastating complication associated with an increased risk of end-stage renal failure, as well as cardiovascular disease and premature death. It has been previously reported that childhood-onset T1D is associated with a 4-fold increase in the overall standardized mortality rate [1].

In both the USA and Europe, approximately 20% of T1D persons develop DN and progress to end-stage renal disease (ESRD). Since T1D often occurs in younger ages, ESRD most often develops at an earlier age, during the most productive years of persons with the disease. Thus, it represents a significant burden to the patients and the society they live in [2, 3]. Moreover, nonalbuminuric DN has been reported to have a prevalence of 2% among people with T1D and chronic kidney disease (CKD) [4, 5].

Nowadays, the screening of DN is based on microalbuminuria (MA) assessment [6], and MA may be found in 12–16% of adolescents with T1D [7]. In early stages, regression to nonalbuminuria is frequently observed [8]. Puberty itself, as well as poor glycaemic control, is an independent risk factor for MA in persons with T1D [9]. However, the diagnostic value of microalbuminuria in DN has recently been challenged by a large number of researchers worldwide, while it has been widely proposed that other biomarkers are needed for the early identification of diabetic renal lesions [2, 5, 10, 11]. Previous studies have provided evidence of several clinical and laboratory predictors for DN, such as increased systolic arterial blood pressure (SAP), even within the prehypertensive range [12], and dyslipidaemia, identifying people at risk for early endotheliopathy and cardiovascular disease (CVD) as well [13]. Among T1D individuals with nephropathy and hypertension, 50% will progress to end-stage renal disease within a decade [6].

The pathophysiologic changes in DN linked to renal function decline are associated with cellular and extracellular derangements in both the glomerular and tubular compartments [14]. Several studies have reported that nonalbuminuric subjects, including prepubertal children with long-standing diabetes, often have glomerular basement membrane (GBM) thickening, mesangial expansion [15, 16], and significant glomerulopathy lesions [17, 18]. Glomerular and renal tubular interstitial injury plays a role in the pathogenesis of DN [19], and various tubular markers have been assessed in the early detection of DN [10, 20]. Among them, neutrophil gelatinase-associated lipocalin (NGAL), first purified and identified in 1993 by Kjeldsen et al., seems to be a promising biomarker [19, 21]. NGAL is a 178-amino acid 25 kDa protein that belongs to the lipocalin protein family. It is primarily produced in renal tubules in response to structural kidney injury [22].

In contrast to conventional markers, such as serum creatinine, blood urea nitrogen, or serum cystatin C (CysC), NGAL is not considered a marker of renal function, but rather reflects structural damage of renal cells. In previous studies, NGAL was reported as effective in the early diagnosis of acute kidney injury (AKI) in several clinical settings [23–25] and was also validated as a significant prognostic factor in cardiovascular morbidity [26]. The association between the early tubular lesions in nonalbuminuric patients with T1D and NGAL was further supported by recently published studies [18, 19, 21].

The aim of this study was to determine the possible predictive role of serum NGAL as a supplementary marker to urinary albumin excretion, in unmasking early renal structural injury, renal function decline, and cardiovascular risk in asymptomatic, normotensive individuals in two different age groups, childhood and adulthood, with various duration of T1D and irrespective of the presence of microalbuminuria.

## 2. Materials and Methods

Two observational cross-sectional prospective long-term follow-up studies took place in two university diabetes centres.

The group A consisted of 57 participants with T1D with a mean age of 13.9 years (SD: 3.1) and a mean diabetes duration of 5.4 years (SD: 3.3) at the time of the evaluation, who were prospectively followed at the Diabetes Centre of the First Department of Pediatrics of the University of Athens, Greece (Table 1) [18].

The group B consisted of 59 young adults with T1D with a mean age of 28.0 years (SD: 4.4) and a mean diabetes duration of 7.4 years (SD: 1.9) at the time of the evaluation, who were prospectively followed for at least 5 years at the Department of Endocrinology of the University Hospital of Linkoping, Sweden (Table 1) [27]. The diagnosis of T1D was based in all participating patients on the presence of the positive titer of at least one of the known circulating, islet-specific, pancreatic autoantibodies related to T1D (autoantibodies against glutamic acid decarboxylase); the 40 K fragment of tyrosine phosphatase (IA2); or insulin antibodies. Regarding group A, eight of the patients presented with microalbuminuria at inclusion in the study, while five of them had known persistent microalbuminuria. In group B, seven out of 59 patients had persistent microalbuminuria at enrolment.

Forty-nine healthy children with a mean age of 10.57 (SD: 6.6) served as controls (group C) (Table 1). Informed consent was obtained from all the participants and their parents before their inclusion in the study. Eighteen healthy adults with a mean age of 27.7 (SD: 4.2) also served as controls (group D).

Both studies were approved by the respective Ethics Committee (Ethics Committee of the Aghia Sophia Children's Hospital in Athens, Greece, and Ethics Committee of the Medical Faculty in Linkoping, Sweden, resp.).

Inclusion criteria for all participants were T1D while exclusion criteria were the presence of active urinary tract infection, glucocorticoid medication, antihypertensive treatment, pregnancy, renal disease, and any chronic disease besides T1D.

Estimated glomerular filtration rate (eGFR) was calculated using the most recently suggested formula by Grubb et al. (eGFR = 130 × Cystatin C^−1.069^ × age^−0.117^ − 7) [28].

Serum and urinary NGAL levels were measured using a commercially available ELISA (Bioporto, Gentofte, Denmark). The reference range in plasma was 37–106 ng/ml, and the intra- and interassay coefficients of variation were 5.6% and 6.4%, respectively.

Cystatin C concentration was determined by an immunonephelometric technique using the BN Prospec nephelometer (Dade Behring, Siemens Healthcare Diagnostics, Liederbach, Germany). The interassay coefficient of variation for the assay was 5.05% and 4.87% at mean concentrations of 0.97 and 1.90 mg/l, respectively, and the reference range in plasma was 0.47–1.09 mg/l.

Statistical analyses were performed using MedCalc, version 12.5 (MedCalc Software, Ostend, Belgium). Correlation analysis was used to determine whether the values of the two variables are associated using Pearson parametric correlation. Student *t*-test was performed (paired samples *t*-test was used in order to test the null hypothesis that the average of the deviations between a series of paired observations is zero when carried out on the same individuals or independent samples *t*-test when performed between the patient and control groups). Multiple regression analysis was used to test the relation between one dependent variable and at least one independent variables. The significance was defined as a *P*value < 0.05, rho, and 95% confidence interval (CI) for the correlation coefficient.

## 3. Results

**Figure 1:**
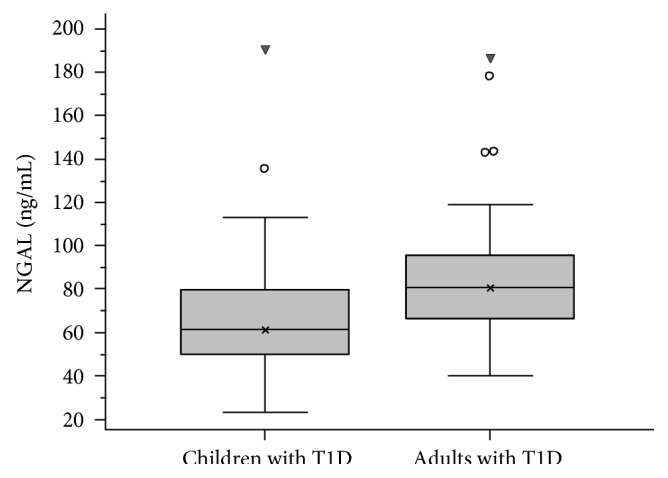
Box plot of NGAL levels in children and adults with T1D. Boxes represent the interquartile range; lines inside the boxes represent the median value; cross represents mean marker; and whiskers represent the lowest and highest observations, respectively. Children group had statistically significant lower median value than the adult group (*P* < 0.001).

**Figure 2:**
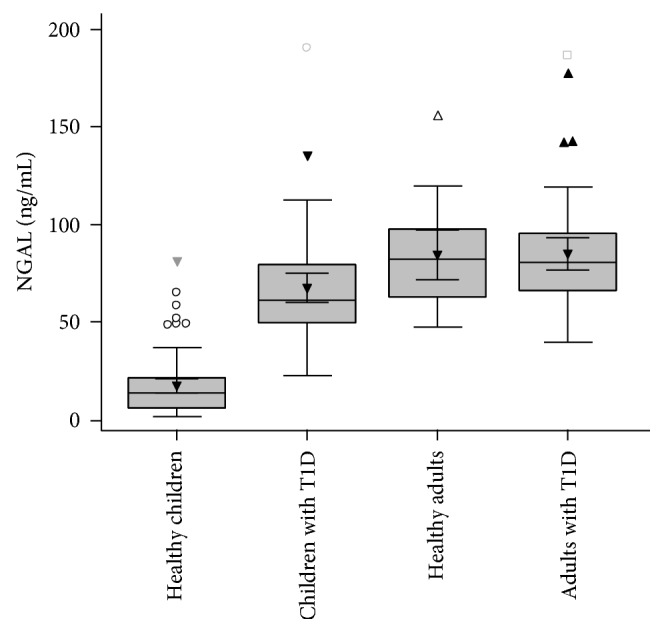
Box plot of NGAL levels. Boxes represent the interquartile range; lines inside the boxes represent the median value; cross represents mean marker; and whiskers represent the lowest and highest observations, respectively. The mean value for NGAL in children with T1D was 67.67 ng/ml (SD: 27.93) while in the group of adults with T1D, it was 85.26 ng/ml (SD: 29.49). Both groups had a significantly higher mean value compared with the two control groups (mean NGAL: 24.69 ng/ml (SD: 15.89) for the children control group (*P* < 0.001) and 76.18 ng/ml (SD: 19.22) for the adult control group (*P* = 0.039)) according to paired *t*-test analysis for independent samples.

The mean value for NGAL in group A was 67.6 ng/ml (SD: 27.9) while in group B, it was 85.2 ng/ml (SD: 29.4) (*P* < 0.001) (Figure 1). Both groups had a significantly higher mean value compared with the two control groups [mean NGAL: 24.6 ng/ml (SD: 15.8) for group C and 76.1 ng/ml (SD: 19.2) for group D], according to paired *t*-test analysis for independent groups (*P* < 0.001 and *P* = 0.039, resp.) (Figure 2).

The mean value for cystatin C in group A was 0.65 ng/ml (SD: 0.1) while in group B, it was 0.71 mg/l (SD: 0.1). Both groups had no significantly higher mean value compared with the two control groups [mean cystatin C: 0.68 ng/ml (SD: 0.1) for group C and 0.74 ng/ml (SD: 0.02) for group D] per paired *t*-test analysis for independent groups.

**Figure 3:**
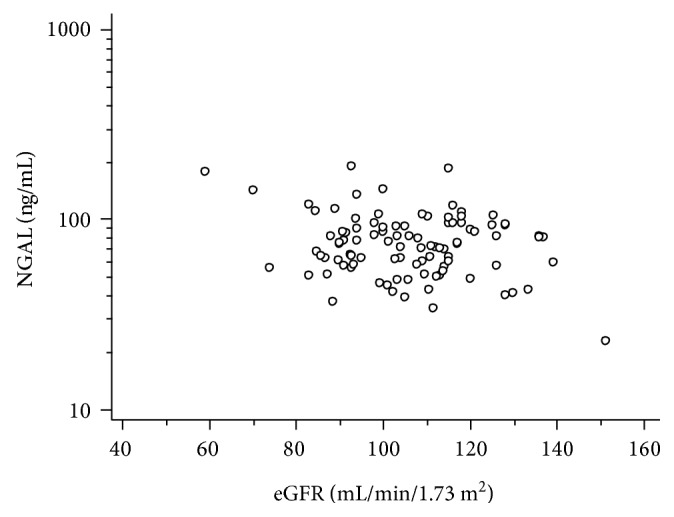
Serum NGAL had a negative correlation with eGFR values according to regression analysis, adjusted for age in both children and adult groups with type 1 diabetes (*F*ratio = 5.93, *P* = 0.0037).

Regression analysis revealed that NGAL had a negative correlation with eGFR in both group A (*r* = −0.501, *P* < 0.001) and group B (*r* = −0,418, *P* < 0.001). Regression analysis of NGAL levels and eGFR values, adjusted for age in both groups A and B with type 1 diabetes, showed a significant correlation between the biomarker and renal function decline (*F* ratio = 5.93, *P* = 0.0037) (Figure 3). No significant correlation was found between NGAL and eGFR in either control groups.

**Figure 4:**
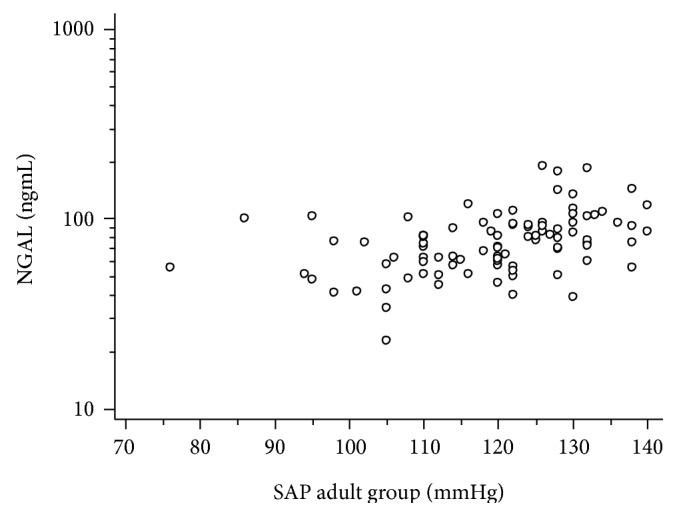
Serum NGAL had a significant positive correlation with systolic arterial pressure for children and adult groups with type 1 diabetes according to regression analysis adjusted for age (*F*ratio = 17.1, *P* = 0.0001).

NGAL was positively correlated with systolic arterial pressure, according to logarithmic curve regression equations in both patient groups (*P* = 0.020 for group A and *P* = 0.031 for group B). In children group A, SAP *z*-score was also analysed and a positive correlation with NGAL was revealed (*P* = 0.009). Regression analysis adjusted for age showed a significant positive correlation of NGAL with systolic arterial pressure in both groups with type 1 diabetes (*F* ratio = 17.1, *P* = 0.0001) (Figure 4). No significant correlation between NGAL and either DAP or DAP *z*-score was noted. NGAL was not significantly correlated with SAP, SAP *z*-score, DAP, or DAP *z*-score in the control groups.

Urinary NGAL was analysed, but no significant correlation with neither SAP nor eGFR was revealed.

Neither microalbuminuria nor HbA1c had any significant correlations with NGAL in either group.

## 4. Discussion

Microalbuminuria has been considered the earliest marker of the development of diabetes nephropathy (DN) and is often linked to established significant glomerular damage, traditionally believed to be the most common lesion in type 1 DN. However, recent studies showed that MA might be transient and not necessarily reflect permanent renal impairment [8]. Further recent reports suggested that pathophysiologic changes in DN include renal function decline associated with both cellular and extracellular derangements in glomerulotubular compartments [17, 18]. Besides, several lines of evidence suggest that early lesions in both glomerular and tubular structures may be present in nonalbuminuric patients. In line with this, cohort studies, including prepubertal children with average diabetes duration of 5–8 years, revealed glomerular base membrane thickening and mesangial expansion [16], while they also disclosed that long-standing nonalbuminuric T1D participants might have significant glomerulopathy lesions [17–19, 21]. Moreover, the prevalence of nonalbuminuric CKD in T1D was recently reported to be 2% and was associated with a higher risk of cardiovascular morbidity as well as all-cause mortality in people with T1D [4, 5].

The increased hyperglycaemia-induced permeability of the glomeruli that is associated with hyperfiltration and microalbuminuria is not debated, but the perception of microalbuminuria being the only marker for diagnosing and excluding the development of diabetic nephropathy needs to be further investigated, to reduce the number of the young T1D normoalbuminuric people who will progress to CKD. According to the National Kidney Foundation (NKF), a patient is considered to be diagnosed with chronic kidney disease (CKD) if he or she presents a GFR < 60 ml/min for three months or more. Alternatively, other ongoing structural or functional renal abnormalities, which can be detected by pathological abnormalities or specific markers, are considered CKD [29]. Besides, early renal function decline, defined as a progressive loss of GFR over time even if it remains within the normal range, is also supported to be associated with DN in T1D [30].

Our study revealed strong negative correlations between serum NGAL and eGFR in both adult and children groups, indicating a significant association with renal function decline. No significant associations between urinary NGAL and eGFR were found in this study, and this finding does not agree with previous reports.

This finding underpins the value of NGAL as a biomarker of early renal damage in T1D, since NGAL had a positive correlation with the duration of T1D, mainly when eGFR was decreased, and this finding may reflect a progress of the early renal structural damage occurring during the disease, even if eGFR remains within the normal range. This finding is probably suggestive that NGAL is associated with the occurrence of ERFD. The number of the participants with T1D who presented microalbuminuria was much lower than the number of patients who presented a decreased eGFR. Moreover, the fact that a percentage of nonalbuminuric patients, who were found to have renal function decline, had higher NGAL levels, while some patients with microalbuminuria had normal NGAL values, may suggest that these two biomarkers, that is, microalbuminuria and NGAL, may reflect other sites of renal injury during the process of DN establishment. The link between the early tubular damage in nonalbuminuric people with T1D and increased NGAL is further supported by recently published studies [15, 17]. The mean value of NGAL was significantly higher in the adult patient group. Besides, regression analysis revealed a correlation between age and the duration of type 1 diabetes in both children and adult. Urinary NGAL was also analysed in the paediatric participants, but no significant correlation with either GFR or SAP was found, and this finding does not agree with previous reports [31].

Systolic arterial pressure has been previously suggested as a predictor of DN [3]. In our study, since it was particularly focused on a young population, we estimated both SAP *z*-score and SAP for children and SAP for adults. We found that in two different age groups with T1D of various disease durations, NGAL correlated positively with increasing systolic arterial pressure, even if the latter remained within the prehypertensive or normal range. It has been previously reported that ambulatory blood pressure modestly rising in T1D patients is associated with the silent phase of DN up to 5 years before microalbuminuria appears [13]. Also, past studies had shown an association between reno-hypertensive diseases and NGAL [31]. Our findings may suggest that the association between NGAL and increasing SAP could reflect the early asymptomatic stages of DN progression in T1D and unravel possible underlying early renal injury. Since we found no similar significant associations in the control groups, our hypothesis is that the findings revealed from the two patient groups reflect the underlying microvascular pathogenesis in type 1 diabetes. Diabetic nephropathy has been linked with biomarker changes consistent with generalized endothelial dysfunction. Markers of endothelial dysfunction and arterial stiffness have also been correlated with MA in diabetic populations [16]. The association between NGAL and increasing SAP may suggest an indirect predictive role of NGAL as a cardiovascular morbidity marker. Undoubtedly, further studies investigating the endothelial dysfunction will further delineate the extent of microvascular damage in DN.

## 5. Conclusions

To our knowledge, this is the first study that aimed at assessing the predictive value of other early markers of renal injury, besides microalbuminuria, such as NGAL in two different age groups of patients with T1D and found similar results. Moreover, a predictive role of NGAL has been demonstrated, as an early marker of diabetes nephropathy and probably asymptomatic cardiovascular morbidity independent of microalbuminuria.

Defining new predictive markers as supplementary tests to urinary albumin excretion for the early diagnosis of microvascular complications in T1D would lead to effective management and treatment approaches in time, which are needed to minimize the rates of severe reno- and cardiovascular as well as all-cause-associated morbidity in young people with T1D. Therefore, these data need to be further confirmed by large-scale longitudinal studies before being integrated into the risk assessment of follow-up of people with T1D.

## Figures and Tables

**Table 1:** Background data, clinical examination, and biochemical measurement results for 57 children with type 1 diabetes, 59 adult patients with type 1 diabetes, and healthy control participants.

Variable	Type 1 diabetes children group (group A)	Healthy control children group (group C)	*P* value	Type 1 diabetes adult group (group B)	Healthy control adult group (group D)	*P* value
Gender	Male 32; female 25	Male 28; female 21		Male 34; female 25	Male 8; female 10	
Age (years)	13.9 ± 1.9 (3.5–18)	10.5 ± 3.3 (3–18)		28.0 ± 2.2 (20–35)	27.7 ± 2.6 (21–36)	
Age at onset (years)	8.5 ± 3.4 (2–15)	NA	NA	8.6 ± 4.1 (1–16)	NA	NA
Diabetes duration (years)	5.4 ± 1.8 (1–14.5)	NA	NA	7.4 ± 0.9 (4.8-9.4)	NA	NA
Mean HbA1c (95% CI for the mean)	7.88%–8.85% (5.8%–14%) or 62.6–73.2 mmol/mol (39.9–129.5 mmol/ mol)	4.4%–4.9% or 25–30 mmol/mol	*P* < 0.001	7.06%–7.88% (4.6%–11.7%) or 53.7–62.6 mmol/mol (26.8–104.4 mmol/ mol)	4.1%–5.2% or 23–33 mmol/mol	*P* < 0.001
BMI (95% CI for the mean)	NA	NA		25.0–27.3 (20.1–40.4)	25.9–30.6 (21.0–30.4)	
BMI *z*-score (95% CI for the mean)	0.2 to 0.8 (−1.0–1.5)	−0.1 to 0.7 (−1.43–1.11)		−0.2 to 0.2 (−1.4–3.3)	−0.1 to 1.2 (−1.3–2.3)	NA
Systolic arterial pressure (mmHg) (95% CI for the mean)	109.81 to 117.18 (76–133)	98.24 to 118.75 (90–119)	*P* = 0.55	121.44 to 126.33 (108–140)	105.35 to 116.31 (100–140)	*P* = 0.272
Systolic arterial pressure *z*-score (95% CI for the mean)	0.02 to 0.58 (−0.9–2.8)	0.01 to 0.39 (−0.4–2.5)	*P* = 0.19	NA	NA	NA
Diastolic arterial pressure (mmHg) (95% CI for the mean)	65.3 to 70.5 (50–85)	56.9 to 67.0 (51–75)	*P* = 0.007	75.8 to 80.0 (60–98)	66.7 to 76.0 (55–90)	*P* = 0.33
Microalbuminuria	8 out of 57	None		7 out of 59	None	
Diastolic arterial pressure *z*-score (95% CI for the mean)	0.14 to 0.5 (−1.1–1.9)	−0.9–0.2 (−1.6–1.5)	*P* = 0.023	NA	NA	NA
NGAL (ng/ml) (95% CI for the mean)	60.2 to 75.0 (22.7–190.3)	20.1 to 29.2 (5.1 to 81.4)	*P* < 0.001	77.0 to 93.4 (39.6–186.5)	67.4 to 84.9 (47.7–109.7)	*P* = 0.039
Cystatin C (mg/l) (95% CI for the mean)	0.23 to 0.92 (0.62 to 0.68)	0.37 to 0.84 (0.63 to 0.78)	*P* = 0.53	0.52 to 1.06 (0.66 to 0.81)	0.38–1.30 (0.68 to 0.75)	*P* = 0.68
eGFR (ml/min/1.73 m^2^) (95% CI for the mean)	98.6 to 105.9 (84.4–151.3)	89.0 to 107.4 (76.4–141.1)	*P* = 0.70	56.6 to 243.3 (113.4–129.5)	72.8 to 170.9 (103.7 to 131.3)	*P* = 0.49

NA: not applicable.
